# Supplementary material for: Effectiveness and challenges of digital tools implementation for enhancing infectious disease surveillance data quality in low- and middle-income countries: A systematic review protocol
Source: PLoS One. 2025 Aug 22;20(8):e0330904. doi: 10.1371/journal.pone.0330904 (PMC12373227; doi:10.1371/journal.pone.0330904)
Supplement: S1 Appendix — (DOCX) [file pone.0330904.s001.docx]

**Search Strategy for the Systematic Review**

A detailed and organized search strategy was created to find pertinent studies regarding the effectiveness of digital surveillance tools/platforms in enhancing the quality of infectious disease surveillance data in Low- and Middle-Income Countries (LMICs). The search was performed across various electronic databases, including PubMed, Scopus, Web of Science, Embase, Cochrane Library, and Google Scholar.

**1. Search Terms and Boolean Operators**

The search strategy was constructed utilizing a mix of Medical Subject Headings (MeSH) terms, keywords, and Boolean operators (AND, OR, NOT) to guarantee a wide-ranging yet accurate retrieval of pertinent literature. The search terms were categorized into four primary concepts:

**Concept 1: Digital Surveillance Tools/Platforms**

- "Digital surveillance" OR "electronic surveillance" OR "mobile health" OR "mHealth" OR "eHealth" OR "telemedicine" OR "electronic health records" OR "EHR" OR "geospatial surveillance" OR "GIS surveillance" OR "artificial intelligence" OR "AI surveillance" OR "big data epidemiology" OR "health informatics" OR "syndromic surveillance" OR "real-time disease surveillance" OR "digital disease detection"
- **Concept 2: Infectious Disease Surveillance**
- "Infectious disease surveillance" OR "communicable disease monitoring" OR "epidemiological surveillance" OR "real-time monitoring" OR "epidemiological monitoring" OR "disease outbreak detection" OR "infectious disease reporting" OR "early warning systems" OR "disease notification" OR "public health surveillance"

**Concept 3: Data Quality Indicators**

- "Data quality" OR "data completeness" OR "data accuracy" OR "data reliability" OR "data timeliness" OR "data validity" OR "data integrity" OR "real-time reporting" OR "data efficiency" OR "standardized data collection" OR "data precision"

**Concept 4: Low- and Middle-Income Countries (LMICs)**

- "Low- and middle-income countries" OR "developing countries" OR "Global South" OR "Africa" OR "Sub-Saharan Africa" OR "Asia" OR "Latin America" OR "resource-limited settings" OR LMICs OR Afghanistan OR Albania OR Algeria OR Angola OR Argentina OR Armenia OR Azerbaijan OR Bangladesh OR Belarus OR Belize OR Benin OR Bhutan OR Bolivia OR "Bosnia and Herzegovina" OR Botswana OR Brazil OR Bulgaria OR "Burkina Faso" OR Burundi OR "Cabo Verde" OR Cambodia OR Cameroon OR "Central African Republic" OR Chad OR China OR Colombia OR Comoros OR "Congo" OR "Costa Rica" OR "Cote d'Ivoire" OR Cuba OR Djibouti OR Dominica OR “Dominican Republic” OR Ecuador OR Egypt OR "El Salvador" OR Eritrea OR Eswatini OR Ethiopia OR Fiji OR Gabon OR "Gambia" OR Georgia OR Ghana OR Grenada OR Guatemala OR Guinea OR Guinea-Bissau OR Guyana OR Haiti OR Honduras OR India OR Indonesia OR Iran OR Iraq OR Jamaica OR Jordan OR Kazakhstan OR Kenya OR Kiribati OR Korea OR Kosovo OR "Kyrgyz Republic" OR Kyrgyzstan OR Laos OR "Lao People's Democratic Republic" OR Lebanon OR Lesotho OR Liberia OR Libya OR Madagascar OR Malawi OR Malaysia OR Maldives OR Mali OR "Marshall Islands" OR Mauritania OR Mauritius OR Mexico OR "Micronesia" OR Moldova OR Mongolia OR Montenegro OR Morocco OR Mozambique OR Myanmar OR Namibia OR Nepal OR Nicaragua OR Niger OR Nigeria OR “North Macedonia” OR Pakistan OR “Papua New Guinea" OR Paraguay OR Peru OR Philippines OR Rwanda OR Samoa OR "Sao Tome and Principe" OR Senegal OR Serbia OR "Sierra Leone" OR "Solomon Islands" OR Somalia OR "South Africa" OR "South Sudan" OR "Sri Lanka" OR "St. Lucia" OR "St. Vincent and the Grenadines" OR Saint Vincent and the Grenadines OR Sudan OR Suriname OR Syria OR Tajikistan OR Tanzania OR Thailand OR Timor-Leste OR Togo OR Tonga OR Tunisia OR turkey OR Turkmenistan OR Tuvalu OR Uganda OR Ukraine OR Uzbekistan OR Vanuatu OR Venezuela OR Vietnam OR "West Bank" OR Gaza OR "Yemen" OR Zambia OR Zimbabwe

**2. Search String for PubMed**

The search terms were combined using Boolean operators as follows:

("Digital surveillance" OR "electronic surveillance" OR "mobile health" OR "mHealth" OR "eHealth" OR "telemedicine" OR "electronic health records" OR "EHR" OR "geospatial surveillance" OR "GIS surveillance" OR "AI-driven surveillance" OR "big data epidemiology" OR "health informatics" OR "syndromic surveillance" OR "real-time disease surveillance" OR "digital disease detection")

AND

("Infectious disease surveillance" OR "communicable disease monitoring" OR "epidemiological surveillance" OR "disease outbreak detection" OR "infectious disease reporting" OR "early warning systems" OR "disease notification" OR "public health surveillance")

AND

("Data quality" OR "data completeness" OR "data accuracy" OR "data reliability" OR "data validity" OR "real-time reporting" OR "standardized data collection")

AND

("Low- and middle-income countries" OR "developing countries" OR "Global South" OR Africa OR "Sub-Saharan Africa" OR Asia OR "Latin America" OR "resource-limited settings" OR "health systems in LMICs" OR lmics OR afghanistan OR albania OR algeria OR angola OR argentina OR armenia OR azerbaijan OR bangladesh OR belarus OR belize OR benin OR bhutan OR bolivia OR "Bosnia and Herzegovina" OR botswana OR brazil OR bulgaria OR "Burkina Faso" OR burundi OR "Cabo Verde" OR cambodia OR cameroon OR "Central African Republic" OR chad OR china OR colombia OR comoros OR "Congo" OR "costa rica" OR "Cote d'Ivoire" OR cuba OR djibouti OR dominica OR "dominican republic" OR ecuador OR egypt OR "El Salvador" OR eritrea OR eswatini OR ethiopia OR fiji OR gabon OR "Gambia, The" OR georgia OR ghana OR grenada OR guatemala OR guinea OR guinea-bissau OR guyana OR haiti OR honduras OR india OR indonesia OR Iran OR iraq OR jamaica OR jordan OR kazakhstan OR kenya OR kiribati OR Korea OR kosovo OR "Kyrgyz Republic" OR "Lao PDR" OR lebanon OR lesotho OR liberia OR libya OR madagascar OR malawi OR malaysia OR maldives OR mali OR "marshall islands" OR mauritania OR mauritius OR mexico OR "Micronesia" OR moldova OR mongolia OR montenegro OR morocco OR mozambique OR myanmar OR namibia OR nepal OR nicaragua OR niger OR nigeria OR "north macedonia" OR pakistan OR papua "New Guinea" OR paraguay OR peru OR philippines OR rwanda OR samoa OR "Sao Tome and Principe" OR senegal OR serbia OR "Sierra Leone" OR "Solomon Islands" OR somalia OR "South Africa" OR "South Sudan" OR "Sri Lanka" OR "St. Lucia" OR "St. Vincent and the Grenadines" OR sudan OR suriname OR syria OR tajikistan OR tanzania OR thailand OR timor-leste OR togo OR tonga OR tunisia OR turkey OR turkmenistan OR tuvalu OR Uganda OR Ukraine OR uzbekistan OR vanuatu OR venezuela OR vietnam OR "West Bank" OR Gaza OR "Yemen" OR zambia OR Zimbabwe)

**3. Database-Specific Adjustments**

PubMed: MeSH terms will be utilized where relevant, and filters will be enforced for peer-reviewed articles.

Scopus/Web of Science: Title, abstract, and keyword searches will be sharpened using field-specific operators.

Embase: Emtree terms will be incorporated to correspond with the database’s indexing system.

CINAHL: Specific CINAHL headings and filters will be enforced.

Google Scholar: Advanced search capabilities will be applied to restrict retrieval to the first 200 peer-reviewed sources and prominent journals.
