# Supplementary material for: Effectiveness and challenges of digital tools implementation for enhancing infectious disease surveillance data quality in low- and middle-income countries: A systematic review protocol
Source: PLoS One. 2025 Aug 22;20(8):e0330904. doi: 10.1371/journal.pone.0330904 (PMC12373227; doi:10.1371/journal.pone.0330904)
Supplement: Attachment — Submitted filename: Rebuttal Letter.docx [file pone.0330904.s004.docx]

Manuscript ID: PONE-D-25-29002

**Review Comment**

In the case of your submission, we note that language restrictions are imposed on the literature searches without adequate justification. There is increasing recognition that the exclusion of studies published in other languages can increase the risk of bias in systematic reviews. We feel that any restrictions should be justified based on scientific reasons rather than resource constraints. As a result, we have concerns about the comprehensiveness of the review and the validity of the conclusions.

**Response**

The criticism is valid. We have, therefore, removed language restrictions from the protocol. Non-English language papers will be translated into English.
